# Pilot study of p62 DNA vaccine in dogs with mammary tumors

**DOI:** 10.18632/oncotarget.2516

**Published:** 2014-09-25

**Authors:** Vladimir Gabai, Franco M. Venanzi, Elena Bagashova, Oksana Rud, Francesca Mariotti, Cecilia Vullo, Giuseppe Catone, Michael Y. Sherman, Antonio Concetti, Andrey Chursov, Anastasia Latanova, Vita Shcherbinina, Victor Shifrin, Alexander Shneider

**Affiliations:** ^1^ CureLab Oncology Inc, Needham MA, USA; ^2^ School of Biosciences and Veterinary Medicine University of Camerino, Camerino (MC) Italy; ^3^ Center of Modern Veterinary Medicine, Kiev, Ukraine; ^4^ Department Biochemistry, Boston University School of Medicine, Boston MA, USA; ^5^ CL Oncology Moscow, Russia

**Keywords:** cancer immunotherapy, vaccine, breast carcinoma, neoadjuvant, p62, canine

## Abstract

Our previous data demonstrated profound anti-tumor and anti-metastatic effects of p62 (sqstm1) DNA vaccine in rodents with various types of transplantable tumors. Testing anti-cancer medicine in dogs as an intermediary step of translational research program provides two major benefits. First, clinical data collected in target animals is required for FDA/USDA approval as a veterinary anti-cancer drug or vaccine. It is noteworthy that the veterinary community is in need of novel medicine for the prevention and treatment of canine and feline cancers. The second more important benefit of testing anti-cancer vaccines in dogs is that spontaneous tumors in dogs may provide invaluable information for human trials. Here, we evaluated the effect(s) of p62 DNA vaccine on mammary tumors of dogs. We found that p62 DNA vaccine administered i.m. decreased or stabilized growth of locally advanced lesions in absence of its overall toxic effects. The observed antitumor activity was associated with lymphocyte infiltration and tumor encapsulation via fibrotic reaction. This data justifies both human clinical trials and veterinary application of p62 DNA vaccine.

## INTRODUCTION

Most of the current research on human cancer mechanisms and treatments are conducted using mice. Mouse models of cancer have several advantages—they can be rapidly propagated, are inexpensive, etc. However, they have essential limitations: while tumors in humans arouse spontaneously, in mice they must be induced by environmental toxins or genetic modifications. In the latter case, it usually involves just one or a few genes, whereas most human cancers are polygenic in origin; therefore, mouse models of cancer are lacking vast gene networks and interactions which are responsible for cancer in humans [1].

Spontaneous cancers observed in dogs have clear advantages as compared to mouse cancers—like human cancers, they occur naturally, are histologically comparable, and respond similarly to anti-cancer therapy [1]. For many gene families, most notably those associated with cancer, the similarities between a dog and human are significantly closer than those between a mouse and human [2]. As the pets live longer due to a better care, the prevalence of cancer in them increases, as also happens in the human population. Importantly, whereas the assessment of disease-free interval or survival in human clinical trials usually takes several years, getting similar information from clinical trials in dogs generally takes much less time, just a few months in some cases.

The latest trend in biotechnology is to test drugs and vaccines in companion animals prior to initiating human clinical trials [3]. For instance, anti-tumor activity of an immune stimulator, liposomal muramyltripeptide phosphatidylethanolamine (L-MPT-PE) was first demonstrated in dogs with osteosarcoma. Later human trials produced remarkably similar results to those of the canine studies [4], which finally lead to approval of L-MPT-PE (MEPACT) for osteosarcoma in children in Europe. Another important example is development of a DNA vaccine for melanoma in dogs; this vaccine (Oncept®) was the first DNA anti-cancer vaccine approved, and its efficiency and safety led to ongoing clinical trials in patients with melanoma.

DNA vaccines, as compared to traditional vaccines, have several advantages: they can induce both humoral and cellular immunity; they are safe, inexpensive and stable; and, they can be easily modified to enhance immune response [5, 6]. Accordingly, there are about 50 ongoing clinical trials which use DNA vaccines for cancer treatment (clinicaltrials.gov).

We have recently developed an anti-cancer DNA vaccine based on p62 (SQSTM1) [7]. The р62 protein is a major player in selective macroautophagy [8] and serves as a signaling hub for several signal transduction pathways, among them NF-kB, TRAF6, MAP kinases, etc. [9], [10, 11]. Importantly, p62 is dispensable for normal tissues, but essential for development and survival of tumors (see [6] for review ). At least in several mouse models studied, knockout of p62 prevented or markedly delayed development of cancer caused by several oncogenes. Furthermore, fully transformed cells do not lose their dependence on p62 since its downregulation causes inhibition of growth or loss of viability [10]. Thus, tumors, in contrast to normal tissues, become “addicted” to p62, a phenomenon known as “non-oncogene addiction” [12]. Importantly, according to data of Oncomine (the largest database of human cancer microarrays) and other reports, at least 10 various types of human cancer have high levels of p62 as compared to normal tissues [10].

Based on these considerations, we have chosen p62 as an antigen for a DNA vaccine, which was evaluated for its anti-tumor effects. In studies of hundreds of animals with allogeneic tumors, p62 vaccine has proven its effectiveness in four types of solid tumors in mice and rats: melanoma, sarcoma, lung and breast carcinomas [7]. More importantly, it also possessed strong anti-metastatic activity in three models of metastatic disease: spontaneous metastases to lung (Lewis lung carcinoma), spontaneous metastases to regional lymph nodes (sarcoma-37), and induced metastases (by I.V. injection) in B16 melanoma. We also found that, at least in case of lung carcinoma and melanoma, p62 vaccine decreased both the number and the size of metastasis, indicating that it suppresses colonization of lung by tumor cells (e.g., formation of micro-metastases), as well as growth of established metastases. In mouse and rat breast carcinomas we also observed, besides tumor growth inhibition, an increase in mean survival by 50-60% ([7] and unpublished data). Based on these rodent data, we decided to move forward to more appropriate clinical models of human cancer, spontaneous canine mammary tumors, and performed a pilot study of efficacy and toxicity of p62 DNA vaccine.

Mammary tumors are the most common tumors of unspayed female dogs with a prevalence of 40% of all tumors, and about half of them are malignant [13]. The major treatment of mammary cancers in dogs is mastectomy, but since the tumors are usually diagnosed at later stages, often surgery cannot be performed and its effect on survival is limited, with medium survival time of only 7-9 months in dogs with stage III tumors [14]. As of now, there is no proven efficacy of any radio- or chemotherapeutic protocol for the treatment of malignant mammary canine tumors, and no immunotherapy for these tumors is available.

Here, we demonstrated that p62-based DNA vaccine is an efficacious and safe treatment of mammary tumors in dogs.

## RESULTS AND DISCUSSION

### Acute Toxicity in Rodents

Before performing chronic toxicity assays in dogs, we have assessed acute toxicity in mice, rats and guinea pigs. Our previous experiments in mice demonstrated that anti-tumor and anti-metastatic activity was the highest at the minimal therapeutic dose (TD) of 0.1-0.15 mg per mouse (i.e., 5-7.5 mg/kg, 3 or 5 times once a week) [7]. Accordingly, the animals were once treated i.m. with 1 or 50 (TD) of the vaccine (1 TD for rats was taken as 7.5 mg/kg, and for guinea pigs – 9.4 mg/kg). Both doses of vaccine were well tolerated and, under autopsy, no apparent pathological changes in organs and tissues were found (data not shown).

### Chronic Toxicity in Rats

A chronic toxicity study was first performed in rats. Rats were administered 1, 5, or 50 TD of the vaccine i.m. once a day for 90 days. All animals survived the treatments and biochemical analysis of their blood did not reveal any toxic effects on heart, kidney and hemostasis (data not shown). There were no negative effects on the rats' behavior or, lipid and carbohydrate metabolism; and, there was no hepatotoxicity except transient mild impairment of liver barrier functions (on day 3). Macroscopic and histological examination after autopsy did not reveal any gross pathological changes (data not shown).

### Chronic Toxicity in Healthy Dogs

Based on the above data about minimal chronic toxicity of p62 DNA vaccine in rodents, we tested its chronic toxicity in dogs. There were no visual signs of intoxications with 5 TD or 50 TD doses (0.1 mg/kg or 1 mg/kg, respectively) during all periods of observation (90 days), although there was a slight (about 10%) decrease in body mass. Hematological analysis revealed a moderate (1.5-2 times) increase in leukocyte numbers, a decrease in lymphocyte numbers, and an increase in thrombocyte numbers (1.5-2 times). Biochemical analysis revealed transient reversible changes in liver functions, similar to rats, and there were no effects on the pancreas and carbohydrate metabolism. Also, no effects on cardiac functions (by ECG) were found. Under autopsy, there were some destructive-dystrophic changes found in kidneys, hypofunction of thyroid and adrenal gland, and a decrease in macrophages in spleen, but these anatomical changes apparently did not cause significant changes in homeostasis (data will be provided on request).

Based on these data, we concluded that the doses of p62 DNA vaccine employed (aproximately 1-10 mg per 10-20 kg animal) can be safely administered to dogs. In contrast to cytotoxic anti-cancer drugs where the therapeutic dose strongly depends on mass and rate of metabolism, such dependence for DNA vaccines is not straightforward. Therefore, for this pilot study we chose doses of 0.75-2.5 mg per dog.

### Toxicity in Tumor-bearing Dogs

Similar to healthy dogs, we did not find apparent drug-associated toxicity in dogs with mammary cancer when the animals were administered vaccine doses ranging from 0.75 to 2.5 mg per dog. During the treatment (3-10 injections once a week), their weight remained unchanged or slightly increased, and their well-being did not deteriorate. No significant differences were found in hematological and biochemical parameters during the treatment as compared to those before treatment (data will be provided on request). Taken together, our toxicity data demonstrates that p62 DNA vaccine is well-tolerated and possesses no apparent toxicity. This conclusion justifies further veterinary and clinical studies of p62 DNA vaccine (see ref [17] for review).

**Table 1:** Patient's Characterization

Pat no.	Breed	Age (yrs)	Type	WHO Stage
1	Yorkshire Terrier	10	Solid carcinoma	T3 N0 M0
2	Badgerer	12	Tubular carcinoma	T3 N1 M0
				
3	Stafford Terrier	14	Papillary carcinoma	T2 N0 M0
				
4	German Shepherd	15	Solid carcinoma	T3 N0 M0
5	German Shepherd	9	Papillary carcinoma	T1 N0 M0
6	German Shepherd	11	Solid carcinoma	T3 N0 M0
7	Mongrel	12	Tubular -papillary carcinoma	T1 N0 M0

### Anti-tumor Activity of p62 DNA Vaccine

**Figure 1:**
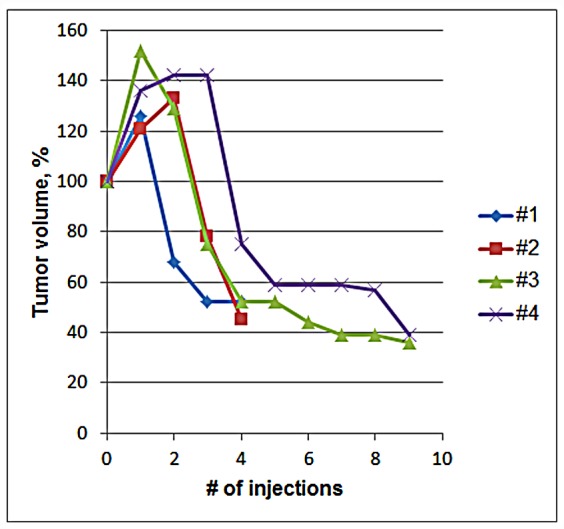
Change in Tumor Volume During Treatment with p62 Vaccine Four mammary tumors of dogs #1-4 (see Tables 1 and 2) were monitored. Initial volumes of tumors were taken as 100%.

The effect of p62 DNA vaccination was evaluated in seven dogs with mammary tumors. We have observed similar responses in all tumors—initially, during first 3-5 injections, tumors become enlarged, but then their size rapidly declined, decreasing in some cases by 50-70% (Fig.1, Table 2). Such an unusual two-phase response is not observed in conventional anti-cancer therapy, but happens in immunotherapy [18] [19]. For instance, tumor volume increases after the first few doses of ipilimumab (anti-CTLA4 antibody) administered in humans, but then it decreases [20]. Importantly, this new pattern of tumor response was associated with favorable survival prognosis, which led to a proposal of improved endpoints of immunotherapy trials [19]. An increase in tumor volume observed initially cannot be due to a tumor growth and propagatoin of cancer cells because it happens too fast. Instead, it is probably associated with local inflammatory immune response (see below).

**Table 2:** Effect of p62 Vaccination on Mammary Tumors in Dogs

Patient #	Dose per Injection, mg	Number of Injections	Initial Tumor Size, cm	Final Tumor Size, cm	Change in Tumor Volume, %	Mastectomy
1	2.5	5	5.9×4.5	4.0×3.8	−50	Yes
2	2.5	5	9.5×4.5	8.1×4.0	−55	Yes
3	2.5	10	5.0×3.5	3.6×2.5	−74	No
4	2.5	10	10×8	6.7×6.1	−71	No
5	1	10	2×2	2×2	0	No
6	0.75	3	8.6×6.2	6.4×3.4	−78	Yes
7	0.75	6	2.2×2.1	2.6 × 1.7	−23	Yes

Apparently, p62 DNA vaccine exerted its effects in two ways: 1) in neoadjuvant settings, it made invasive and non-resectable tumors resectable (patients #1, 2, 6, 7), and 2) if mastectomy was impossible because of health concerns or declined by an owner, the treatment completely stopped tumor growth for the entire duration of the observation period (patients #4, 5). So far, only one dog (#3) demonstrated relapse of primary tumor after 6 months, and it is now undergoing another round of treatment with the vaccine; all other dogs are currently either tumor-free (after mastectomy) or have had their tumors stabilized for 4-22 months (mean = 10 months). Such prolonged stabilization of tumors without their disappearance is also characteristic of immunotherapy, which, in contrast to chemo- or radiotherapy, have long-lasting effects due to immunological memory. It may mean, for instance, that immune response arrests tumor growth, but it is not sufficient for complete tumor eradication. Smaller size tumor(s) remaining in the body may not be critical for dog's well-being even if they never regress completely. We are still collecting the data on long-term effects of p62 vaccine and overall survival of the dogs.

### Insights in Mechanism of Anti-tumor Activity of p62 DNA Vaccine

**Figure 2:**
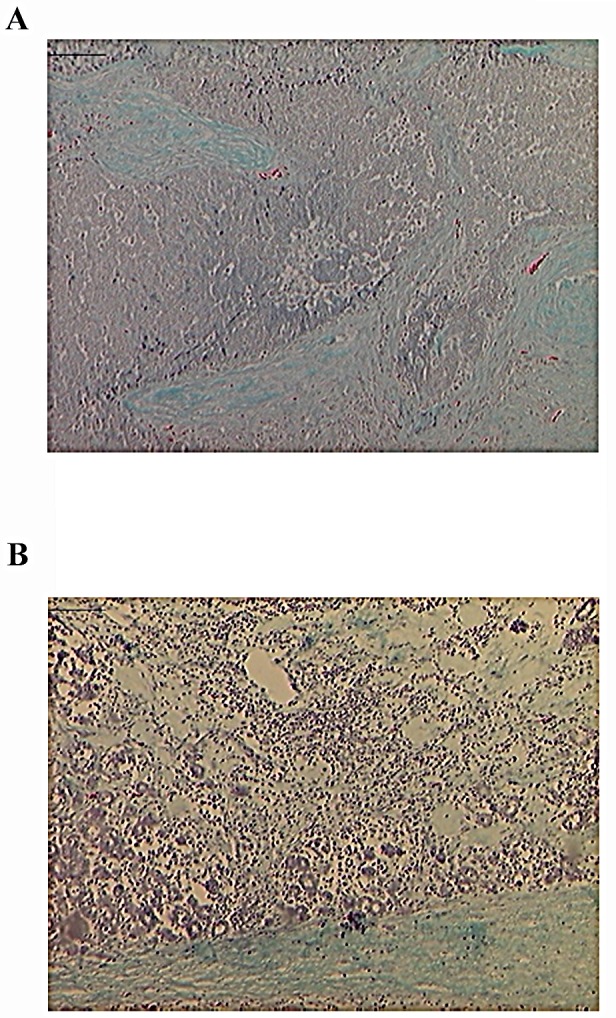
Mononuclear Inflammatory Infiltrates After p62 Vaccination Green-blue - collagen /connective tissue; dark-blue dots – monocytes (Gomori staining). A) 1st biopsy was taken before p62 vaccination; B) 2d biopsy – after p62 vaccination. Magnification - 10x.

In several mouse tumor models (е.g., [21]) and in human cancers, in particular breast cancer, lymphocyte infiltration is regarded as a favorable prognostic factor indicating enhanced host's anti-tumor immune response [22-24]. Since we observed an apparent increase in tumor volume during the first several injections, we tested whether this was caused by lymphocyte infiltration associated with inflammatory immune response. Indeed, analysis of biopsies before and after treatment with p62 DNA vaccine demonstrated significant infiltration of monocytes within the neoplasms (Fig.2).

**Figure 3:**
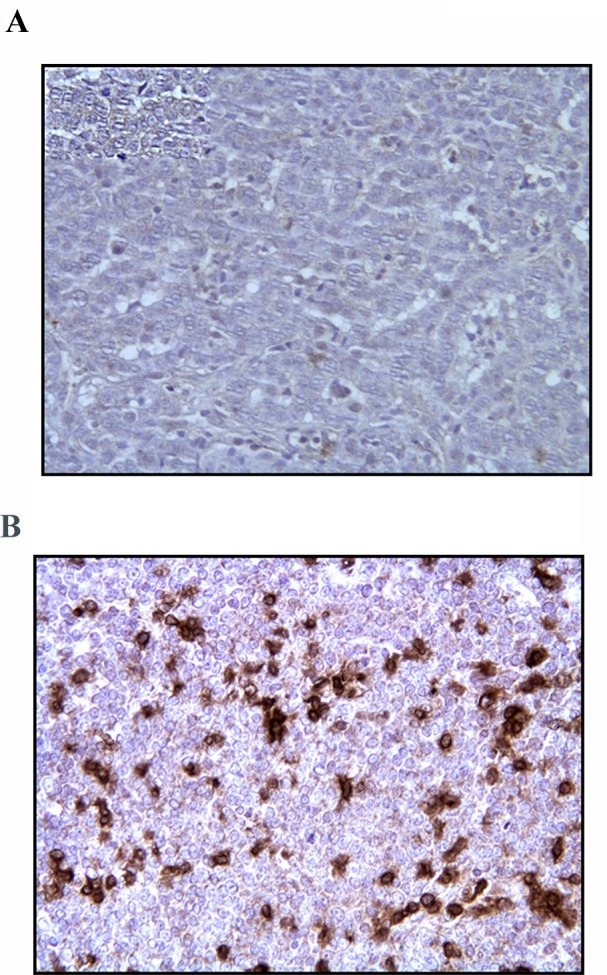
T Cells Infiltration of Mammary Tumor After p62 Vaccination A) Tumor biopsy was taken before treatment; B) resected tumor after the treatment (dog#6). Staining with rat anti-CD3 monoclonal Ab was made as detailed in Materials and Methods. Magnification – 20x.

**Figure 4:**
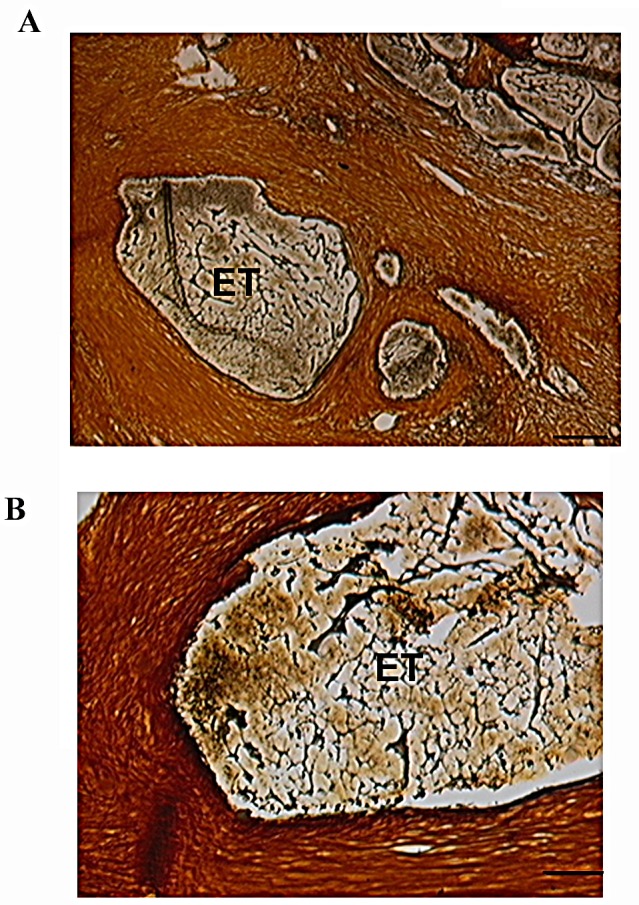
Tumor Islands Surrounded by Stroma After p62 Vaccination Silver impregnation staining in section of resected tumor. Tumor islands surrounded by stroma: brown is a connective tissue, black is reticular fibers, and yellow is collagen. Calibration bars: A, 100μm; B, 50 μm. ET, encapsulated tumor.

Also, histological sections after mastectomy obtained from two tumors treated with p62 DNA vaccine were subjected to immune staining with anti-CD3 antibody specific for T-lymphocytes (including cytotoxic CD8+ lymphocytes) and compared to the same tumor's biopsies before the treatment. As shown in Fig.3, p62 DNA vaccination resulted in a drastic accumulation of CD3+-positive cells in the tumor as compared to the control without the treatment. Finally, we found that p62 DNA vaccination induces profound changes in the extracellular matrix of mammary neoplasms. As shown in Fig.4, silver-stained sections of tumors [25] consistently displayed strong and highly organized tissue proliferation surrounding and slitting the original neoplastic mass into smaller tumor islands. This localized desmoplastic reaction is due to the vaccination because it is not observed in retrospective review of dozens of untreated dogs with mammary adenocarcinoma (unpublished data and ref [26] ). However, it remains to be revealed how a thick layer of stroma surrounding a tumor contributes to prevention of the tumor growth and spreading.

In addition to the data presented above, we observed a positive effect of p62 DNA vaccine in feline mammary tumors, and in other tumors of dogs (e.g., lymphoma) (manuscript in preparation). Duration of a progression-free survival and clinical applicability of the p62 DNA vaccine to different nosologies and stages of disease will be assessed quantitativly in follow-up studies.

Previously, pre-operative chemo- or radiotherapy were used as neoadjuvants in patients with early stage breast cancer. Neoadjuvant therapy, which could be used for pre-treatment of inoperable tumors to make these tumors resectable, constitutes an important unsatisfied niche. To the best of our knowledge, our study is the first report of neoadjuvant application of an antitumor vaccines (or immunotherapy) in humans or dogs. We observed clear benefits of neoadjuvant immunotherapy for the dogs which did not have adjuvant therapy as an available option. In future studies, we may asses effect(s) of combination(s) of p62 vaccines with conventional aduvants.

## CONCLUSIONS

1. p62 DNA vaccine demonstrated no overall toxicity in healthy and tumor-bearing dogs, and it is well-tolerated by animals without visible side effects.

2. p62 DNA vaccine demonstrated anti-tumor activity in canine spontaneous mammary tumors leading to tumor shrinkage and/or stabilization.

3. Along with our previous data on anti-tumor and anti-metastatic activity of p62 DNA vaccine in allogeneic tumors in rodents and a comprehensive set of pre-clinical data, these results justify further clinical development of p62 DNA vaccine as a veterinary medicine and its advancement into human clinical trials.

## MATERIALS AND METHODS

### p62 Vaccine

Human p62 (SQSM1, isoform 1) was cloned in pcDNA3.1 vector. Plasmid DNA was produced by Aldevron (ND, USA). Quality of plasmid preparation was assessed by DNA agarose electrophoresis, by sequencing and by functional assay for p62 activity (Gabai et al, in preparation).

### Toxicology Study in Healthy Dogs

Chronic toxicity was tested on beagles (3 males and 3 females, weight 12-14 kg). The dogs were administered daily i.m. injections of either 5 therapeutic doses (TD) (3 dogs) or 50 TD (3 dogs) (0.1 mg/kg or 1 mg/kg, respectively) for 90 days (total dose 9 mg/kg or 90 mg/kg, respectively). During the treatment they were monitored by hematological and biochemical analyses of blood and for cardiac functions by EKG. Dogs were euthanized 3 or 30 days after full course of injections and their autopsies were was performed. This study was performed according to standards of Good Laboratory Practice and guidelines of Russian Ministry of Health.

### Patients and Treatment

Assessment of the therapeutic effect was performed in three veterinary clinics: Center of Modern Veterinary Medicine, Kiev (Ukraine), University of Camerino (Italy), and Municipal Veterinary Clinic, St. Petersburg (Russia). A total of seven dogs, all females, of different breeds and ages 9 to 15 yr old (mean = 12 yr) were enrolled in the study (Table 1). All of them had histologically confirmed diagnosis of breast carcinoma with WHO stages I-III, progressive disease, and no options for treatment or other treatment options were declined by the owners. P62 vaccine was administered i.m. once a weak at the doses of 0.75, 1.0 or 2.5 mg for 3-10 weeks (Table 1). During the treatment, blood was collected for biochemical analysis, and the sizes of tumors were measured manually with calipers. Also the weight and overall well-being of patients were monitored. All the treatments were performed with full consent of the owners.

### Tumor Specimens, Histochemistry and Immunohistochemistry

Biopsies (Trucut) were performed in all dogs before treatment to establish initial diagnosis. In two of them (patients #6 and #7), a second biopsy, along with samples from resected tumors were collected. Standard histochemical analysis of connective tissue, Giemsa, Masson's Trichome staining, and silver impregnation was carried out as detailed elsewhere [15].

For immunohistochemistry, sections of the tumors were deparafinazed and processed as previously described [16]. CD3+T cells were stained with rat anti-human CD3 monoclonal antibody (Serotec) and Elite ABC-peroxidase KitsStandard (Vectasain).
